# Supplementary figures and images for: Recurrent disease progression networks for modelling risk trajectory of heart failure
Source: PLoS One. 2021 Jan 6;16(1):e0245177. doi: 10.1371/journal.pone.0245177 (PMC7787457; doi:10.1371/journal.pone.0245177)

S1 **Fig.** Heart failure frequency distribution in our CHD dataset.

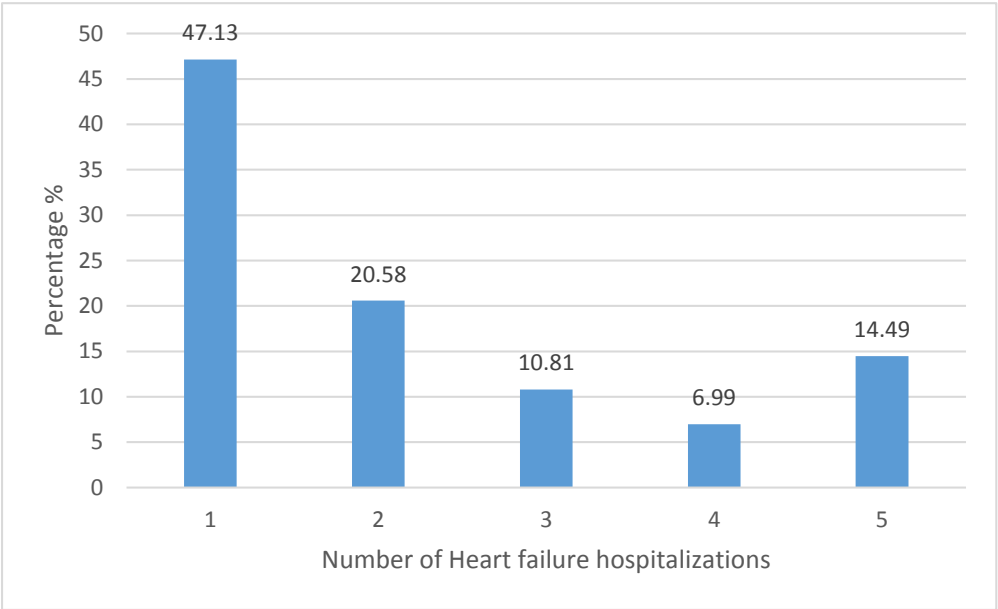

Supplement: S1 Fig — (PDF) [file pone.0245177.s001.pdf]
