# Supplementary material for: Recurrent disease progression networks for modelling risk trajectory of heart failure
Source: PLoS One. 2021 Jan 6;16(1):e0245177. doi: 10.1371/journal.pone.0245177 (PMC7787457; doi:10.1371/journal.pone.0245177)

S2 **Fig.** Precision and recall scores for the conservative threshold. Each RNN model was given 15 years of co-morbidities changes and heart failures, starting from the age of 40. The precision (a) and recall (b) scores as a function of years were then evaluated for each of the 3 RNN on the test patients.

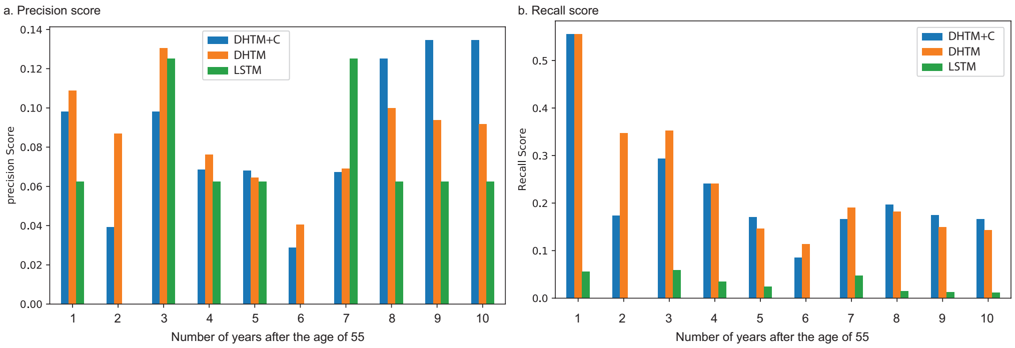

Supplement: S2 Fig — Each RNN model was given 15 years of co-morbidities changes and heart failures, starting from the age of 40. The precision (a) and recall (b) scores as a function of years were then evaluated for each of the 3 RNN on the test patients. (PDF) [file pone.0245177.s002.pdf]
