# Supplementary material for: Recurrent disease progression networks for modelling risk trajectory of heart failure
Source: PLoS One. 2021 Jan 6;16(1):e0245177. doi: 10.1371/journal.pone.0245177 (PMC7787457; doi:10.1371/journal.pone.0245177)

S3 **Fig.** ROC and Precision-recall in predicting next time heart failure onset. We compared 5 different methods namely logistic regression, SVM, and three recurrent neural network approaches, which are RNN with LSTM recurrent unit, Deep Heart Trajectory Model (DHTM), and Single-time-step Model (STM). The overall precision-recall curve and the "zoom-in" view on the recall at 0.2 are displayed.

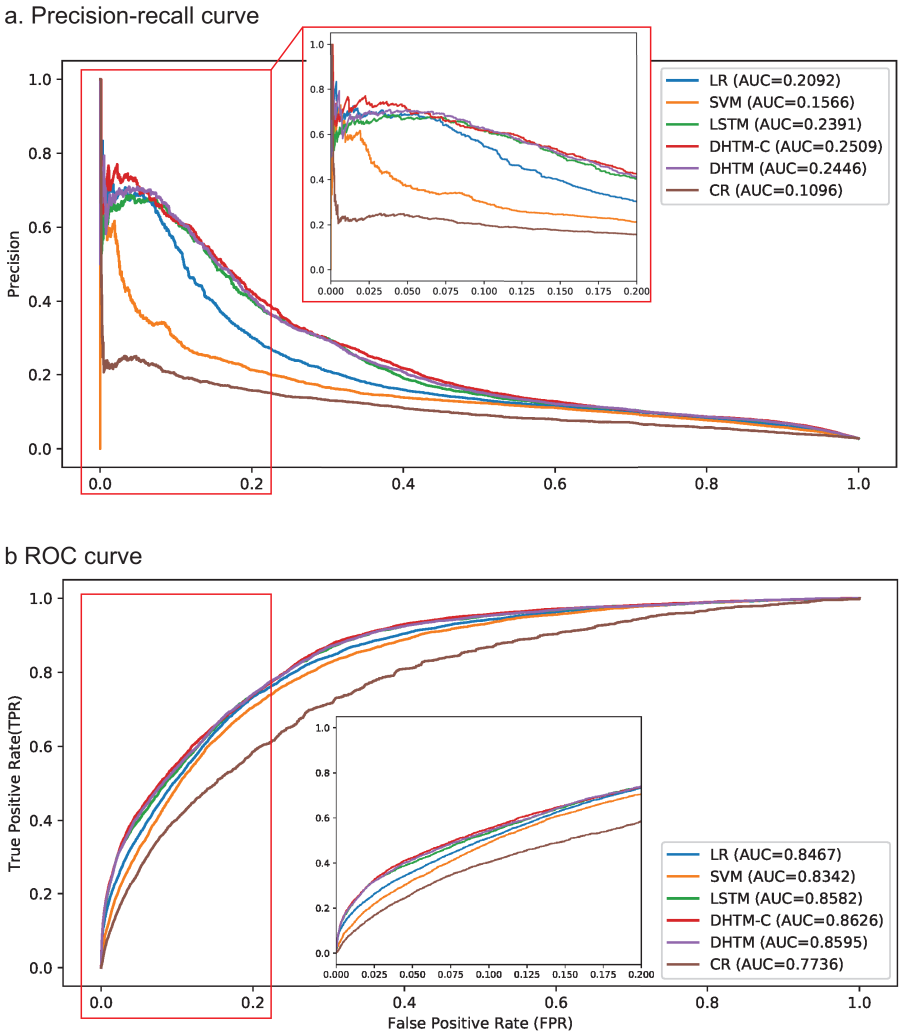

Supplement: S3 Fig — We compared 5 different methods namely logistic regression, SVM, and three recurrent neural network approaches, which are RNN with LSTM recurrent unit, Deep Heart Trajectory Model (DHTM), and Single-time-step Model (STM). The overall precision-recall curve and the “zoom-in” view on the recall at 0.2 are displayed. (PDF) [file pone.0245177.s003.pdf]
