# Supplementary material for: Recurrent disease progression networks for modelling risk trajectory of heart failure
Source: PLoS One. 2021 Jan 6;16(1):e0245177. doi: 10.1371/journal.pone.0245177 (PMC7787457; doi:10.1371/journal.pone.0245177)

S4 **Fig.** Hinton Plots on each RNN model. Hinton plots for the 3 RNN model weights in the first input-hidden layer. We plot from top to bottom the weights of the dense layer for DHTM+C, DHTM, and LSTM, respectively. For each panel, The rows are the 20 co-morbidity variables and the columns are the 64 hidden units. whiteand black color indicate positive and negative weights, respectively. The size of the squares is proportion to the magnitude of the connection weights between the input units and the hidden units.

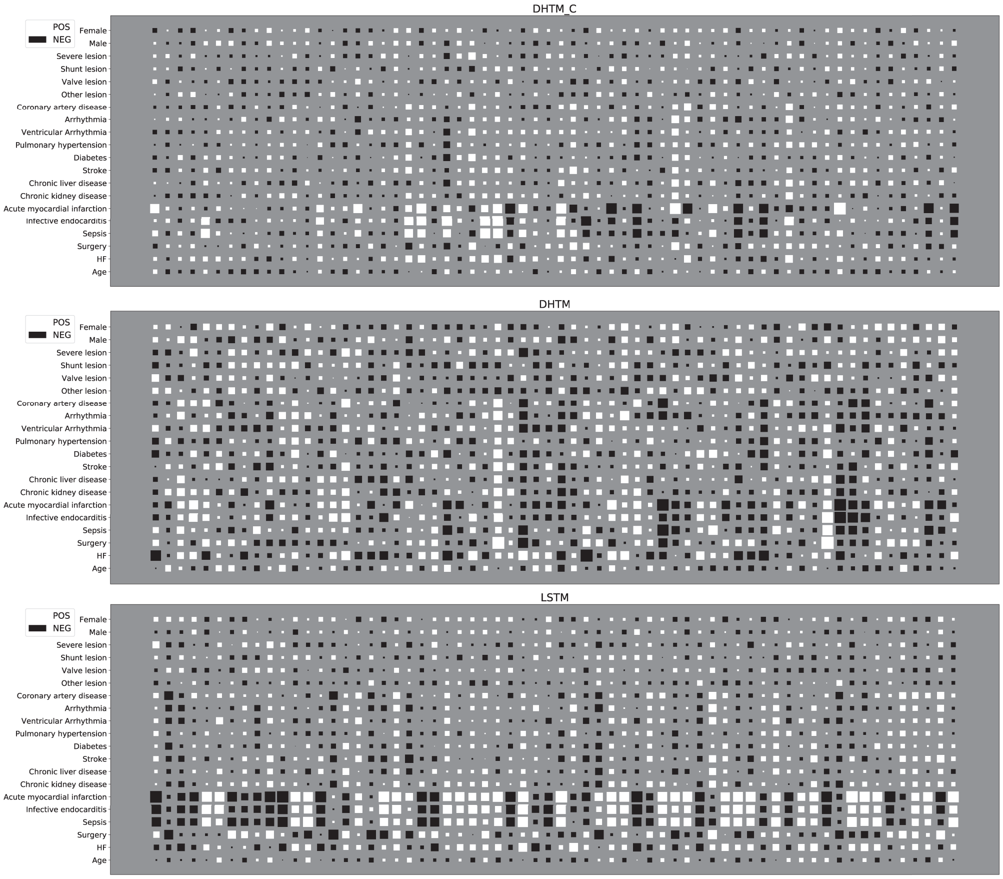

Supplement: S4 Fig — Hinton plots for the 3 RNN model weights in the first input-hidden layer. We plot from top to bottom the weights of the dense layer for DHTM+C, DHTM, and LSTM, respectively. For each panel, The rows are the 20 co-morbidity variables and the columns are the 64 hidden units. white and black color indicate positive and negative weights, respectively. The size of the squares is proportion to the magnitude of the connection weights between the input units and the hidden units. (PDF) [file pone.0245177.s004.pdf]
