# Supplementary material for: Recurrent disease progression networks for modelling risk trajectory of heart failure
Source: PLoS One. 2021 Jan 6;16(1):e0245177. doi: 10.1371/journal.pone.0245177 (PMC7787457; doi:10.1371/journal.pone.0245177)

S5 **Fig.** F1 Scores for our three recurrent architectures. Each model was given 15 yearsof co-morbidities changes and heart failures, starting from the age of 40. The F1 scoreas a function of years was then evaluated for each of the 3 RNN on the test patients.

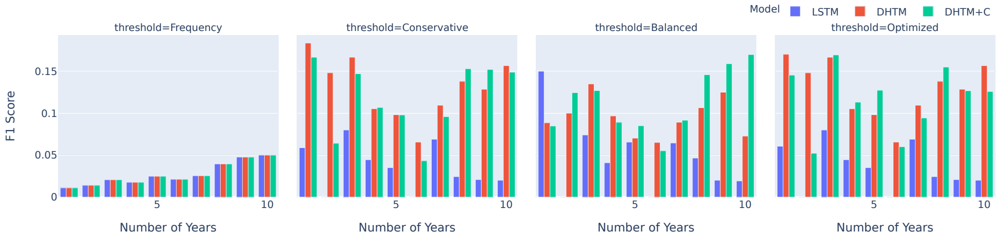

Supplement: S5 Fig — Each model was given 15 yearsof co-morbidities changes and heart failures, starting from the age of 40. The F1 scoreas a function of years was then evaluated for each of the 3 RNN on the test patients. (PDF) [file pone.0245177.s005.pdf]
