# Supplementary material for: Recurrent disease progression networks for modelling risk trajectory of heart failure
Source: PLoS One. 2021 Jan 6;16(1):e0245177. doi: 10.1371/journal.pone.0245177 (PMC7787457; doi:10.1371/journal.pone.0245177)

S6 **Fig**. Distributions of risk scores predicted by each of our RNN models. Whereas LSTM collapses at 0, both the DHTM+C and DHTM models spread the risk score over a greater range. For this reason, a different threshold is used for every model, as displayed in **S2** Table

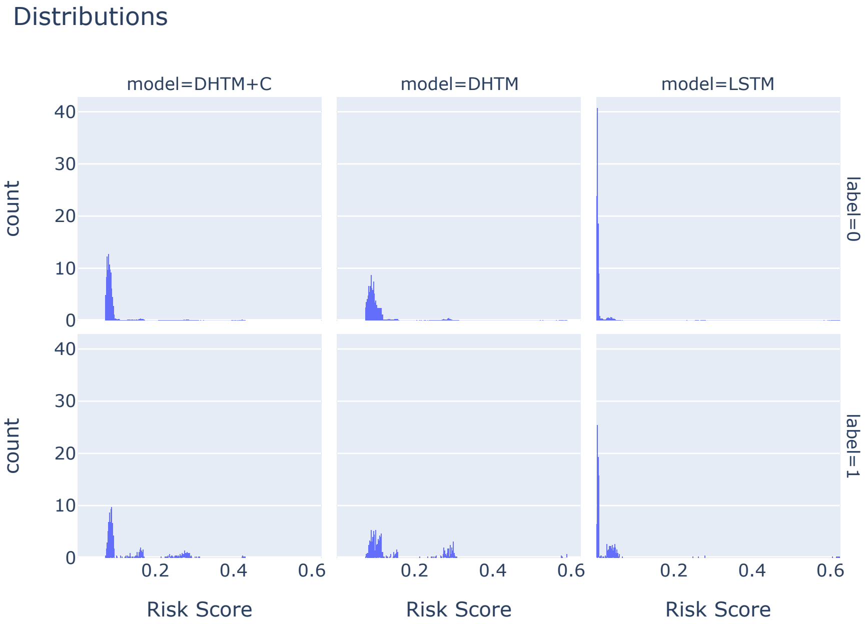

Supplement: S6 Fig — Whereas LSTM collapses at 0, both the DHTM+C and DHTM models spread the risk score over a greater range. For this reason, a different threshold is used for every model, as displayed in S2 Table. (PDF) [file pone.0245177.s006.pdf]
