# Supplementary material for: Recurrent disease progression networks for modelling risk trajectory of heart failure
Source: PLoS One. 2021 Jan 6;16(1):e0245177. doi: 10.1371/journal.pone.0245177 (PMC7787457; doi:10.1371/journal.pone.0245177)

S7 **Fig.** Loss history of the co-morbities prediction task in the DHTM+C model. The model was trained for 30 epochs, but quickly decreased to 0.0005 due to the sparsity of the labels.

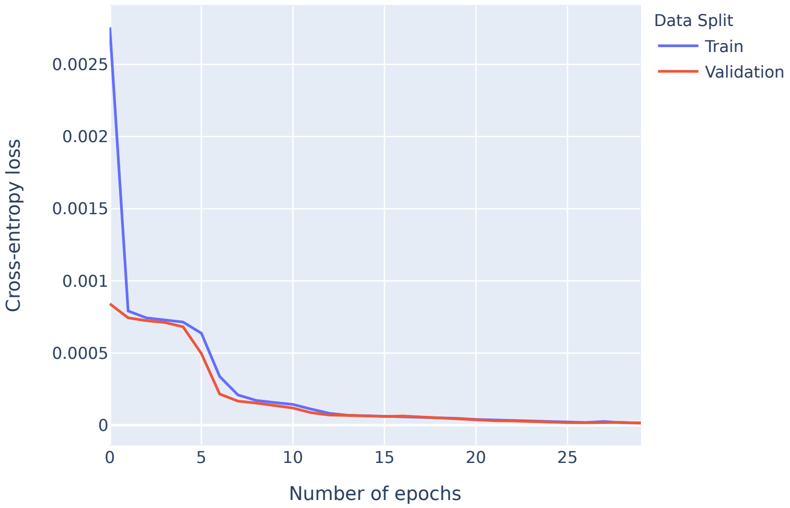

Supplement: S7 Fig — The model was trained for 30 epochs, but quickly decreased to 0.0005 due to the sparsity of the labels. (PDF) [file pone.0245177.s007.pdf]
