# Supplementary material for: Recurrent disease progression networks for modelling risk trajectory of heart failure
Source: PLoS One. 2021 Jan 6;16(1):e0245177. doi: 10.1371/journal.pone.0245177 (PMC7787457; doi:10.1371/journal.pone.0245177)
Supplement: S1 Table — (PDF) [file pone.0245177.s008.pdf]

S1 **Table.** Patient distribution in congenital heart defects

Lesion type	Congenital heart defects	Frequency	Percent%
Severe	Endocardial cushion defect	310	3.38
Severe	Tetralogy of Fallot	232	2.53
Severe	Univentricular heart	86	0.94
Severe	Transposition complex including: complete and congenitally corrected	148	1.62
Severe	Truncus arteriosus	47	0.51
Severe	Ebstein anomaly	18	0.20
Severe	Hypoplastic left heart syndrome	39	0.43
Shunts	Atrial septal defect	1191	13.00
Shunts	Ventricular septal defect	516	5.63
Shunts	Patent ductus arteriosus	85	0.93
Shunts	Aortic coarctation	89	0.97
Shunts	Unspecified defect of septal closure	233	2.54
Valvular	Anomalies of the pulmonary artery	38	0.41
Valvular	Anomalies of the pulmonary valve	208	2.27
Valvular	Congenital tricuspid valve disease	32	0.35
Valvular	Congenital aortic stenosis	570	6.22
Valvular	Congenital aortic insufficiency	263	2.87
Valvular	Congenital mitral stenosis	18	0.20
Valvular	Congenital mitral insufficiency	627	6.84
Valvular	Anomalies of great veins	55	0.60
Unspecified	Other unspecified anomalies of the heart	2657	29.01
Unspecified	Other unspecified anomalies of the aorta	696	7.60
Unspecified	Other unspecified anomalies of the circulation including PDAS and coarctation	716	7.82
Unspecified	Unspecified	286	3.12
