# Supplementary material for: Recurrent disease progression networks for modelling risk trajectory of heart failure
Source: PLoS One. 2021 Jan 6;16(1):e0245177. doi: 10.1371/journal.pone.0245177 (PMC7787457; doi:10.1371/journal.pone.0245177)
Supplement: S2 Table — A threshold is applied for every patients and every time step for a given model. (PDF) [file pone.0245177.s009.pdf]

S2 **Table.** The value of the thresholds for each of the four methods. A threshold is applied for every patients and every time step for a given model.

| **Model** | **Frequency** | **Balanced** | **Conservative** | **Optimized** |
|---|---|---|---|---|
| **LSTM** | 0.00129098 | 0.0599885 | 0.11658455 | 0.181 |
| **DHTM** | 0.00129098 | 0.1166855 | 0.16353217 | 0.219 |
| **DHTM+C** | 0.00129098 | 0.1283573 | 0.18698153 | 0.252 |
