# Supplementary material for: Recurrent disease progression networks for modelling risk trajectory of heart failure
Source: PLoS One. 2021 Jan 6;16(1):e0245177. doi: 10.1371/journal.pone.0245177 (PMC7787457; doi:10.1371/journal.pone.0245177)
Supplement: S3 Table — (PDF) [file pone.0245177.s010.pdf]

S3 **Table.** Complex levels of cardiac surgical operations in patients with congenital heart disease.

**Complex level**	**Cardiac surgical operations**
**I**	Shunt closure; Shunt take down; ASD closure; Patent ductus ligation; Arrhythmia surgery; Pacemaker procedures; ICD procedures
**II**	VSD closure; Ruptured sinus of Valsalva repair; Coronary artery fistula repair; Cavopulmonary anastomosis; Systemic arterial to PA shunt; ASD/VSD creation/enlargement; Blalock procedure; Pulmonary veins repair; Atrioventricular canal repair; PA banding and debanding; Coarctation repair; Vascular ring repair; Valvular and aortic surgery - Right heart; Pulmonary artery repair; Pulmonary valve surgery; Tricuspid valve surgery; Coronary artery bypass
**III**	Fontan procedure; Aortic arch repair; Fallot repair; Left ventricle to aorta repair; Atrial switch; Transannular patch repair; Valvular and aortic surgery - Left heart; Aortic valve surgery; Ross/ Konno procedure; Bentall procedure; Aortic root replacement; Aortic aneurysm repair; Aortic dissection repair; Mitral valve surgery
**IV**	Norwood procedure; Konno/Ross-Konno procedure; Unifocalization PA anastomosis Rastelli procedure; Arterial switch; Anomalous coronary correction; Transplants/mechanical hearts; Heart and lung transplant; Mechanical heart
