# Supplementary material for: Recurrent disease progression networks for modelling risk trajectory of heart failure
Source: PLoS One. 2021 Jan 6;16(1):e0245177. doi: 10.1371/journal.pone.0245177 (PMC7787457; doi:10.1371/journal.pone.0245177)
Supplement: S4 Table — Each RNN model was given 15 years of co-morbidities changes and heart failures, starting from the age of 40. They then predicted the next 10 years of HF events. (PDF) [file pone.0245177.s011.pdf]

S4 **Table.** Table of area under the receiver operating characteristic curve (AUROC) area under the precision-recall curve (AUPRC) for every year in the trajectory prediction. Each RNN model was given 15 years of co-morbidities changes and heart failures, starting from the age of 40. They then predicted the next 10 years of HF events.

**Years**	**AUPRC**			**AUROC**		
	**DHTM+C**	**DHTM**	**LSTM**	**DHTM+C**	**DHTM**	**LSTM**
**1**	**0.0591**	0.0583	**0.0869**	0.8128	0.8141	**0.8201**
**2**	**0.0464**	**0.0602**	0.0467	**0.8027**	0.7384	0.7675
**3**	0.0704	**0.0739**	**0.0830**	**0.7548**	0.7448	0.7472
**4**	**0.0399**	**0.0410**	0.0398	0.7376	**0.7524**	0.7519
**5**	**0.0327**	0.0314	**0.0378**	**0.6609**	0.6388	0.6456
**6**	0.0258	**0.0279**	**0.0235**	**0.6135**	0.6103	0.6089
**7**	**0.0552**	**0.0585**	0.0545	0.6305	0.6395	**0.6404**
**8**	**0.0677**	0.0670	0.0631	**0.6555**	0.6291	0.6306
**9**	**0.0732**	0.0673	0.0714	**0.6757**	0.6705	0.6709
**10**	**0.0697**	0.0610	0.0677	**0.6291**	0.6024	0.6031
