# Supplementary material for: Recurrent disease progression networks for modelling risk trajectory of heart failure
Source: PLoS One. 2021 Jan 6;16(1):e0245177. doi: 10.1371/journal.pone.0245177 (PMC7787457; doi:10.1371/journal.pone.0245177)
Supplement: Attachment — Submitted filename: DHTM_PLoS1_Response.pdf [file pone.0245177.s012.pdf]

Response to Reviewers

Editor

In addition to the reviewers comments, I would like you to consider reporting the age at diagnosis.

Our response: we thank the Editor for this suggestion. We have included patient distribution in the age at the first heart failure hospitalization into **Section 3.1 page 3, line 65**:

"*Regarding age at the first HFH, the mean and median values were 61.80 and 68.26, respectively, with an interquartile of 54.82 – 77.13.*"

You should also have your article read by a clinician that use the correct medical terminology (see comments reviewer #2). Furthermore, it is not entirely clear to me if you only included patients with congenital heart diseases as cause of HF.

Our response: we thank the reviewer for the suggestion. We have had the manuscript reviewed by a clinician and made corrections on medical terminologies. Given that the paper aims to model heart failure trajectories in patients with congenital heart disease (CHD), the study population is CHD patients with at least one heart failure hospitalization as described in **Section 3.1 page 3 line 64**:

"*For the HF study, only patients with at least one HF event were selected.*"

It seems to be the case. Therefore, you should be clear also in the title that you refer to this kind of patient with HF. They may be very different from other patients with HF. You also need to define the lesions of CHD.

Our response: Indeed, CHD patients with HF are different from other patients with HF in disease mechanism and treatment. We have extended the **Conclusion** section (page 10, line 330-335) to emphasize this difference.

"CHD patients have different disease mechanisms and treatment for heart failure from the general population. Thus, the clinical interpretation of the prediction models for heart failure could not be generalized to the general population."

We have added the definition of CHD lesion in **Section 3.1 page 3 line 76** as well as **Supplementary Table 1** (shown below for ease of reading this letter) in **Supporting Information** for the details of the CHD lesion distribution.

"All patients were assigned 1 or 2 CHD diagnoses using a previously described and validated hierarchical algorithm (Marelli et al., 2007). Severity of CHD was classified on the basis of anatomic diagnosis into 4 types: severe, shunts, valvular, and unspecified CHD lesions (Table S1). Severe lesions were with the highest probability of being associated with cyanosis at birth among the 4 lesion types. Patient distribution in specific CHD lesions is shown in Supplemental Table S1."

I also miss a description of the number and time course of events in the patient group.

Our response: we have included a figure to show the distribution of the number of heart failure events and time course. The following description has been added to **Section 3.1 page 3 line 70**:

*"Among the 9160 CHD patients with at least one heart failure hospitalization (HFH), nearly half (47.13%) had one HFH. One fifth had two HFHs and one tenth had three HFHs. About 15% of the patients had 5 or more HFHs (**Supplemental Figure S1**). Regarding age at the first HFH, the mean and median values were 61.80 and 68.26, respectively, with an interquartile of 54.82 – 77.13."*

Figure S1. Histogram for the distribution of number of heart failure hospitalizations

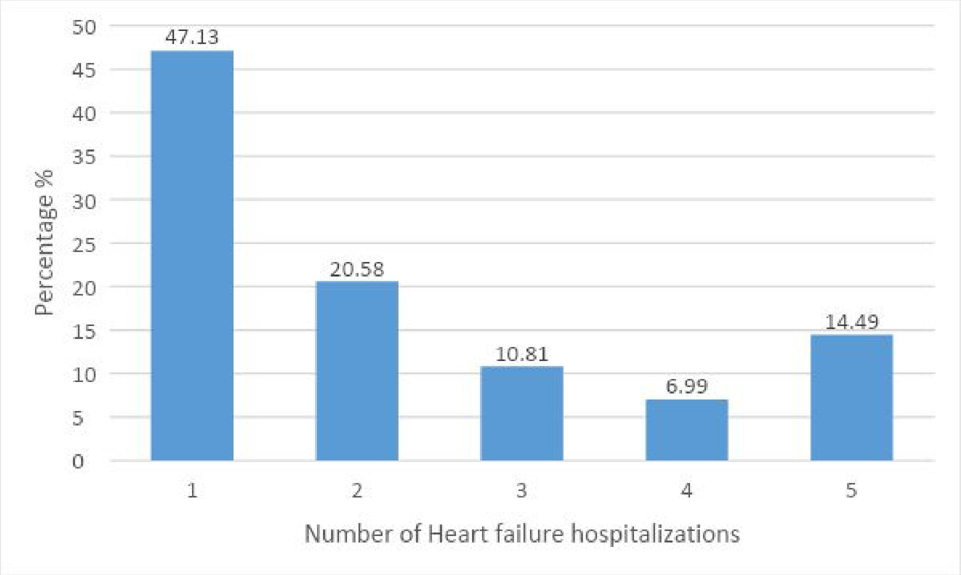

Reviewer #1

This manuscript reports a study of predicting HF events using RNN models. The authors designed two RNNs, i.e., DHTM and DHTM+C, both of which are based on the GRU architecture. Then the authors trained the models on an EHR dataset and evaluated the prediction performances. These models were also compared to the baseline models including logistic regression, SVM and a standard LSTM model. The results are promising, and the manuscript is well organized.

Our response: Thank you very much for the positive comments and constructive suggestions. Please see our point-by-point response below.

However, there are a number of problems with the manuscript, and there are also some concerns with the study:

1) The statements on p. 5, lines 146-149 implicitly contained the following two points:

a) In the DHTM model, the residual connections and layer normalization were added in order to lessen the vanishing gradient problem.

b) Adding CMs as targets of prediction in addition to HF in the DHTM+C model can lessen the vanishing gradient problem of the DTHM model.

However, both of the two points are problematic:

For a), the authors already described on p. 4 line 112 that the vanishing gradient problem is dealt with by the update gate in GRU models. Since DHTM model is based on GRU, why is vanishing gradient still a problem for DHTM model, which led to the addition of residual connection and layer normalization?

Our response: We thank the reviewer for pointing out this confusion. By design, GRUs are capable of mitigating vanishing gradients caused by backpropagation *through all of the time points* [22]. However, in order to build deeper GRU models, we also needed to ensure that our models can correctly backpropagate gradients *through all of the layers*. For the DHTM model, we introduced residual connections and layer normalization to mitigate vanishing gradients across layers. Residual connections were implemented in the transformer architecture in deep models with up to 12 hidden layers [26]. Layer normalization mitigates the effect of exploding/vanishing gradients caused by the magnitude of the recurrent units. For the DHTM+C model, because of the more frequent feedback produced by the prediction loss of the 3 CM and HF, we did not implement the residual connections and layer normalization. We have extended **Methods Section 4.2.4** to describe the approaches.

- [22] Chung J, Gulcehre C, Cho K, Bengio Y. Empirical Evaluation of GatedRecurrent Neural Networks on Sequence Modeling. arXiv:14123555 [cs]. 2014;
- [26] Vaswani A, Shazeer N, Parmar N, Uszkoreit J, Jones L, Gomez AN, et al.Attention is all you need. In: Advances in neural information processing systems;2017. p. 5998–6008

For b), why adding more targets of prediction can lessen the vanishing gradient problem?

Our response: For the DHTM+C model, we add the prediction loss of the comorbidity variables at every time step as an auxiliary loss term to the prediction loss of HF. This ensures that the model is updated more frequently by the gradients of the total loss (sum of the HF loss and the CM loss) at every time step. As a result, the model is able to model the progression of HF risk and learn a stable representation of the changes in CMs at every time step.

We added the above clarification to our revised manuscript. Please see **Section 4.2.1**

2) The description of the objective functions (section 4.2.3) is confusing. Specifically,

a) In Eq. (1), the subscripts of y and yhat are missing.

b) In Eq. (2), both the summation and subscripts are missing.

Our response: Thank you for letting us know about this point of confusion. We have corrected the equations 1 and 2 with the subscripts and summation.

c) It seems that both Eqs. (1) and (2) are used only the prediction of HF events. Since the DHTM+C model predicts multiple outcomes - both HF and CM events, what is the objective function defined for DHTM+C?

Our response: Since HF and each CM are binary values, it's possible to represent both HF and all the CMs as a single multi-output binary cross entropy objective, where y is a multi-label vector and y-hat is a sigmoid-activated vector representing the predicted progression in HF risk and CMs. Thus, both eqs (1) and (2) are applicable to both DHTM (where the y and y-hat are a scalar for a given time step) and DHTM+C (where y and y-hat are vectors).

Thank you for pointing out the confusion about the two equations, we expand our explanation concerning how those objectives are computed for each model.

Please see **Section 4.2.3**

3) What is the objective function for the LSTM model? Is it the same as those in section 4.2.3, or a different one? If not the same, then the difference between the predictions of the LSTM model and the predictions of the DHTM/DHTM+C models may be explained by the difference in the objective functions.

Our response: Yes we use the same objective function for the LSTM model. We have extended a sentence in **Section 4.3** for clarification:

"Additionally, we evaluated the standard LSTM model, which does not use the more advanced residual connections and layer normalization techniques as in our DHTM model, ***but uses the objective function as in Eq (2)****"*

4) For evaluation, precision and recall are included in addition to other metrics. However, directly comparing precisions (or recalls) of different models is unfair, since one can be optimized at the cost of the other. The authors should compare F-scores instead.

Our response: Thank you for the suggestion. It is indeed difficult to visualize the precision and recall charts separately. We replaced it with a bar figure of the F1 scores in the revised manuscript (**Figure 3**) also displayed below, and moved the precision and the recall charts to the appendix.

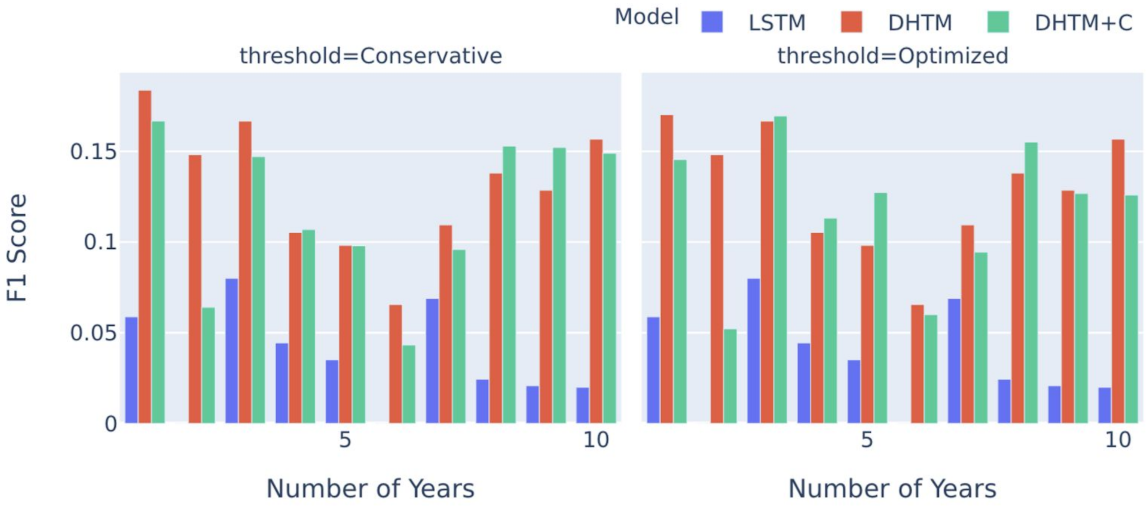

Fig 3. F1 Scores for our three recurrent architectures. Each model was given 15 years of co-morbidities changes and heart failures, starting from the age of 40. The F1 score as a function of years was then evaluated for each of the 3 RNN on the test patients. The conservative (a) and optimized (b) methods were used to compute the thresholds. For a comparison between all four methods, see figure S5

5) Precision and recall depend on a choice of thresholds. Why is the threshold chosen as the average of probabilities?

Our response: Thank you for this question. Choosing the thresholds that can generalize well to unseen patients is indeed an important issue.To this end, we tested 4 thresholds. Let $p_1, p_2, \cdots p_k$ be the scores computed by a model for the k time points for all of the HF events across all of the time points over all of the patients in the training set. Let $n_1, \cdots, n_m$ be the scores for the m time points across all of the non-HF events. In total, there are $m+k$ time points in the training dataset. Then, we computed the means of p's and n's as $\overline{p} = (p_1 + \cdots + p_k) / k$ and $\overline{n} = (n_1 + \cdots + n_m) / m$, respectively. The four thresholds are defined below and evaluated for each method in terms of F1 Score (**Supplementary Figure S5**). Unless mentioned otherwise, the thresholds were chosen based on the full training data (i.e., 75% of the total patients).

- **Frequency threshold** is calculated as the total number of positive labels divided by the total number of patients: $m / (m+k)$;
- **Conservative threshold** is calculated as the average risk score of the true HF events in the training set: $\overline{p}$;
- **Balanced threshold**. To increase the sensitivity in non-resource constrained situations, we can calculate the midpoint between the average risk score of the true HF events, and the average risk score of the non-HF timesteps: $(\overline{p} + \overline{n}) / 2$;
- **Optimized threshold**. which iteratively finds the threshold via a grid search that maximizes the F1 Score based on the validation set. This is a commonly used threshold in the ML community. The threshold is found via a grid search that maximizes the F1 Score based on the validation set. We trained each model on the training set, which is 63.75% of the total patients. We set aside 11.25% of the total patients as the validation set to select the best threshold that gives the highest validation F1 score for each method. Therefore, we used in total 75% of the data to train and choose thresholds. We then evaluated each model based on their optimized threshold on the test set, which is 25% of the total patients.

In the initial submission, we used the **conservative threshold** since it is most relevant in a resource constrained context and clinically meaningful as the model identifies the high risk patients that are at need for immediate medical interventions.

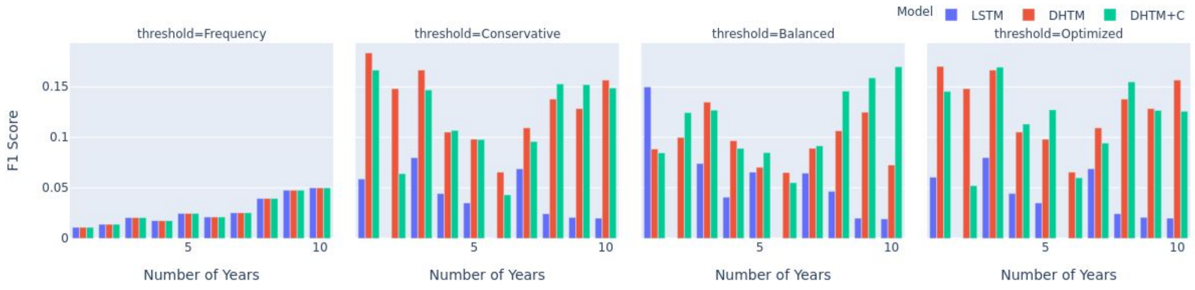

In the case of the **frequency threshold**, we observe a very poor performance across all time steps for every model, which indicates a poor fit for our task and is likely caused by the large number of negative labels in the training set. For both the **balanced** and the **conservative** thresholds, we observe that the DHTM and DHTM+C outperform the LSTM baseline model for trajectory modeling, with the exception of the first time step for the balanced threshold. The performance of using the **optimized** threshold determined by the validation set is similar to the **conservative** threshold determined by the training set.

Considering that LSTM's F1 Score immediately drops to 0 in the subsequent time step and that the model is unable to achieve a stable F1 Score afterwards, it is reasonable to assume that the LSTM is overfitting on correctly predicting the next time step rather than the subsequent ones. As we recall, DHTM introduced architectural changes (such as residual connections and layer normalization) and DHTM+C added an auxiliary training objective (comorbidities progression modeling). Those changes enabled them to model temporal features with increased depth and to explicitly detect changes in comorbidities before predicting risk of HF. As a result, we observe a consistent F1 score throughout the entire trajectory for both DHTM and DHTM+C rather than prioritizing predicting the next event, which accounts for the decrease in performance for only the first time step.

We added the **balanced**, **conservative**, and **optimized** thresholds in **Section 4.4**, and the exact values of the threshold as **Supplementary Table S2**. We displayed in the main text **Figure 3** with the F-1 scores using **conservative** and **optimized** thresholds and **Figure S5** in the Supplementary Information for F-1 scores on all of the four thresholds. We also summarized the explanations above in the revised manuscript to clarify the use of thresholds.

6) Did the authors choose different thresholds for different models, or one threshold for all models?

Our response: As described above, all of the thresholds are method-independent. The first 3 thresholds (i.e., frequency, conservative, balanced) were chosen based on the training data and the optimized thresholds were chosen based on the validation set for each model. To further justify this, we also examined the distributions of predicted scores by each method, as shown in this figure (added as **Supplementary Figure S6**):

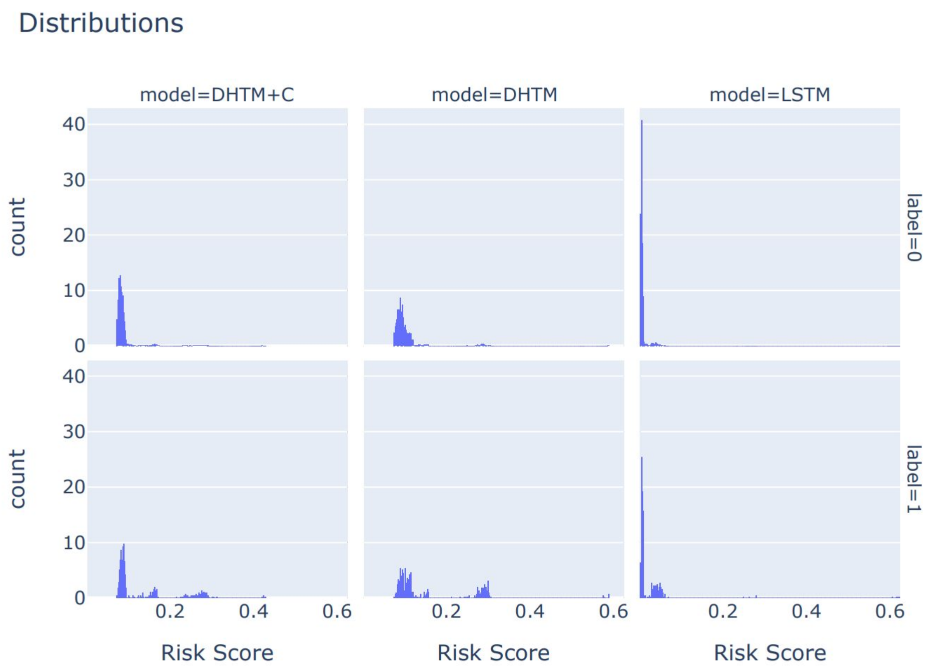

The threshold is a function of the risk scores of each model. There is no single threshold that works the best for all of the methods. The table below (added as **Supplementary Table S2**) indicates all of the thresholds determined for each method:

**Model**	**Frequency**	**Balanced**	**Conservative**	**Optimized**
**LSTM**	0.00129098	0.0599885	0.116584554	0.181
**DHTM**	0.00129098	0.11668553	0.16353217	0.219
**DHTM+C**	0.00129098	0.128357365	0.18698153	0.252

7) For LSTM model, what are the AP score and the precision at 1% recall? These two are reported for DHTM and DHTM+C models in section 5.1.

Our response: we thank the reviewer for pointing out this lack of clarification. The term AUPRC and AP were used interchangeably, which created confusion. Thus, we changed all of the mention of “AP” to “AUPRC” and correctly referenced the **Supplementary Table S4** in the **Supporting Information**.

8) In section 5.1, it is stated that "Cox regression achieved the lowest prediction accuracy at 10% AP." What does "prediction accuracy at 10% AP" mean? How did the other models perform on this metric?

Our response: as explained in the previous response, we have used AUPRC for assessing the model performance. The sentence that raises confusion has been corrected.

9) S2 Fig is exactly the same as Fig 3. Based on the description of S2 Fig, it seems that S2 Fig points to the wrong figure. In addition, it seems that the AUROCs should be reported in S2 Fig, but the description of S2 Fig only mentions AP and AUPRC.

Our response: We thank the reviewer for pointing out the error. We have corrected the figures. We now report the AUROC and AUPRC in **Supplementary Table 4**.

**Years**	**AUPRC**			**AUROC**		
	**DHTM+C**	**DHTM**	**LSTM**	**DHTM+C**	**DHTM**	**LSTM**
**1**	0.0591	0.0583	**0.0869**	0.8128	0.8141	**0.8201**
**2**	0.0464	**0.0602**	0.0467	**0.8027**	0.7384	0.7675
**3**	0.0704	0.0739	**0.0830**	**0.7548**	0.7448	0.7472
**4**	0.0399	**0.0410**	0.0398	0.7376	**0.7524**	0.7519
**5**	0.0327	0.0314	**0.0378**	**0.6609**	0.6388	0.6456
**6**	0.0258	**0.0279**	0.0235	**0.6135**	0.6103	0.6089
**7**	0.0552	**0.0585**	0.0545	0.6305	0.6395	**0.6404**
**8**	**0.0677**	0.0670	0.0631	**0.6555**	0.6291	0.6306
**9**	**0.0732**	0.0673	0.0714	**0.6757**	0.6705	0.6709
**10**	**0.0697**	0.0610	0.0677	**0.6291**	0.6024	0.6031

10) The authors evaluated AUPRC and AUROC for the trajectory prediction for each year over 10 years, but I do not see these AUCs reported in Results.

Our response: we thank the reviewer for pointing out the error. We have replaced the figure by the table above (included in **Supplementary Table 4**).

Based on the AUPRC and AUROC, the performances are in general similar among the three RNN models. This is attributable to the sparse HF events throughout the patient's history. As mentioned above, we focused on the F-1 scores based on the empirically determined thresholds for each method.

11) On p. 7, line 199, it is stated that, for Task 2, the authors evaluated AUPRC and AUROC, while on the same page, line 206, it is stated that, for both tasks, the authors evaluated AUROC and AP. Then what is exactly evaluated for Task 2, AUPRC or AP or both? This is confusing.

Our response: The term AUPRC and AP were used interchangeably, which created confusion. To remain consistent, we changed all references to AP into AUPRC.

12) For DHTM+C, are the evaluations done for only the HF prediction, or for all the CM predictions as well?

Our response: We verified during training that DHTM+C was correctly learning from the CM labels by reducing the loss function for CMs, as shown in this figure (added as **Supplementary Figure S7**):

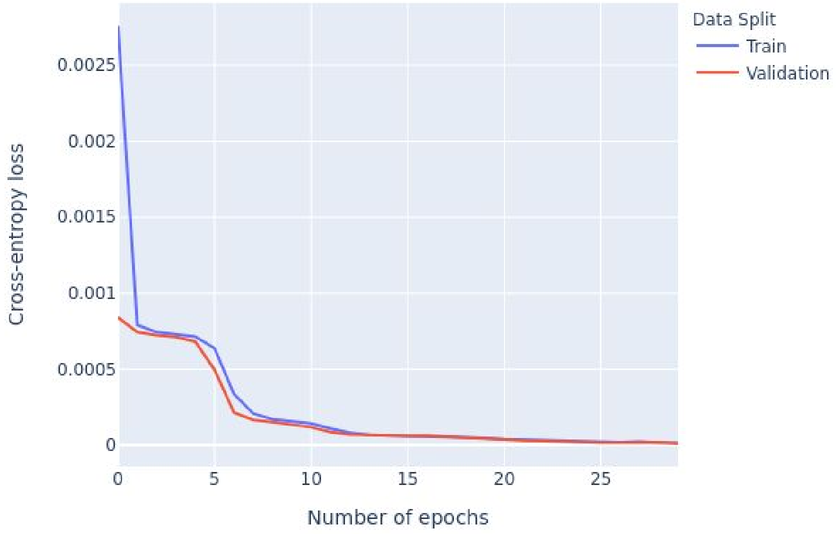

We have added a sentence in the **Methods Section** (page 6, line 161) to clarify this.

Reviewer #2

The authors of this study have made two heart failure prediction models (DHTM and DHTM+C) with recurrent neural networks based on GRU architecture. The dataset is derived from the EHR documented Quebec Congenital Heart Disease where they chose to model the prediction for heart failure in case of congenital heart disease, because heart failure origination from CHD would be better to predict. They chose data of adults from the age of 40 in order to exclude the disease profile of children. They ended up with data of 8093 patients of which they used 75% to train the neural network, and 25% to validate it. They used 16 covariates in the DHTM+C model to not only predict the heart failure events, but also onset of comorbidities and their effect of heart failure events. They compared the result of their GRU based RNN with baseline models LSTM, Logistic Regression, Support Vector Machine and Cox regression. The results showed that their two models outperformed the baseline models. These results are promising. However, the model is inclined to predict for no change for comorbidities, which could improve with further development.

1. On line 19 you state “Compared to HF induced by shock or chemicals...”, what is meant by shock? Shock is mostly a result of heart failure, not a cause.

Our response: We thank the reviewer for noticing this wrong statement. We have removed the inaccurate statement about shock and chemicals.

2. On line 46 and 51 the listings are hard to read when using a period (.). Maybe suggest using semicolon (;).

Our response: Thank you for letting us know about this. We corrected the listings for all of section 2 using semicolons.

3. On line 64 you describe HF events and history. How is heart failure and heart failure event defined? (LVEF <35%? or <40% or <50%? HFpEF? HFrEF?) You state that you include patients with at least one HF event. What kind of events? Acute heart failure events with need of admission? Were there patients with heart failure (<35% LVEF) but without "events"? If so, why were only patients with at least one event included? Do you think data bias could result from this?

Our response: we define heart failure events as the hospitalizations with heart failure being the admission and/or discharge diagnoses. Given our data source is an administrative database, we have no clinical date to assess LVEF. We have added the following sentence to clarify HF events (**page 3, line 70**):

*“W*e define heart failure events as the hospitalizations with heart failure being the admission and/or discharge diagnoses.”

4. On line 72 you state "surgery of four different complex levels". Could you give an example of "severe" and "other" to make this more clear?

Our response: we have extended the Methods section and added a new table to clarify the classification of surgeries (**Section 3.1,** page 3, line 92-96):

"We used surgical operations from four complexity categories: I, II, III, IV based on an adaptation of the Aristotle complexity score [20] and published by our group [21]. ***Supplemental Table S3*** *displays the complex levels of the cardiac surgeries. For example, shunt closure is classified as complex level I while anomalous coronary correction is at complex level IV. Due to the very low frequency of surgeries records across all the four complex levels, we combined the four types of surgeries into a single variable to overcome the sparsity to have a meaningful estimation of its effect on HF."*

- [20] Jacobs J.P. Mavroudis C. Jacobs M.L. et al. Lessons learned from the data analysis of the second harvest (1998-2001) of the Society of Thoracic Surgeons (STS) Congenital Heart Surgery Database. Eur J Cardiothorac Surg. 2004; 26: 18-37
- [21] Ionescu-Ittu, Raluca, et al. "Valvular operations in patients with congenital heart disease: increasing rates from 1988 to 2005." The Annals of thoracic surgery 90.5 (2010): 1563-1569.

5. Table 1: Suggestion: make P values more readable by writing < 0.01 or < 0.01 or 0.01 or 0.34 for example.

Our response: Thank you for the suggestion. We have updated **Table 1** (pasted below) as suggested.

Co-Morbidity	HF+<br>N=9160	HF-<br>N=74953	Log<br>Ratio	p-value
Death	3557 (38.8%)	5066 (6.8%)	2.52	< 0.001
CHD Lesion				
Severe lesion	970 (10.6%)	8530 (11.4%)	-0.1	0.0171
Shunt lesion	2025 (22.1%)	40445 (54.0%)	-1.29	< 0.001
Valve lesion	1811 (19.8%)	11754 (15.7%)	0.33	< 0.001
Other lesion	4354 (47.5%)	14224 (19.0%)	1.32	< 0.001
Acute myocardial infarction	2724 (29.7%)	2714 (3.6%)	3.04	< 0.001
Coronary artery disease	5447 (59.5%)	6986 (9.3%)	2.67	< 0.001
Arrhythmia	4155 (45.4%)	3699 (4.9%)	3.2	< 0.001
Ventricular arrhythmias	992 (10.8%)	846 (1.1%)	3.26	< 0.001
Pulmonary hypertension	2771 (30.3%)	2182 (2.9%)	3.38	< 0.001
Infective endocarditis	637 (7.0%)	837 (1.1%)	2.64	< 0.001
Diabetes	2799 (30.6%)	3490 (4.7%)	2.71	< 0.001
Stroke	2215 (24.2%)	3610 (4.8%)	2.33	< 0.001
Chronic liver disease	557 (6.1%)	765 (1.0%)	2.57	< 0.001
Chronic kidney disease	2788 (30.4%)	1717 (2.3%)	3.73	< 0.001
Sepsis	988 (10.8%)	1923 (2.6%)	2.07	< 0.001

Table 1. Comparison in selected demographic and clinical characteristics between CHD patients with and without HF. The p-values were generated by binomial test with background rate set to 9160/(9160+74953)=0.109.

6. On line 314 you state that "the model is inclined to predict for no change for these CM". So was the model not successful in predicting new onset om CM? Are there results on this?

Our Response: The observation that our model is inclined to predict for no change for the CMs is consistent with our data which contains only disease diagnoses but no clinical data for marking disease progression. We have chosen the most powerful CMs among candidate predictors based on literature and our previous publications on heart failure (References 1-3 listed below). Among them, the majority are lifelong conditions since birth such as congenital heart defects or chronic conditions since the first onset such as diabetes. For these CMs, there is no change in the data since birth or the first diagnosis. As for the acute CMs that might have new onsets, the rate of recurrent events is very low. For example, only 3.03% of the patients have recurrent sepsis and 1.67% have recurrent infectious endocarditis. We have extended the **Discussion section (page 11, line 343)** to describe this data limitation and point out the need to include clinical data in future studies.

"Thus, the model is inclined to predict no change for these CM. This is consistent with our data which contains only disease diagnoses but no clinical data for marking disease progression ..."

References:
1. Cohen, Sarah, et al. "Risk prediction models for heart failure admissions in adults with congenital heart disease." *International Journal of Cardiology* (2020).
2. Wang, Fei, et al. "Heart failure risk predictions in adult patients with congenital heart disease: a systematic review." *Heart* 105.21 (2019): 1661-1669.
3. Wang, Fei, et al. "Risk of readmission after heart failure hospitalization in older adults with congenital heart disease." *International journal of cardiology* 320 (2020): 70-76.

7. Description of S3 Fig.: I assume in the sentence "While and black color indicate positive and negative weights, respectively.", the word "While" should be "White".

Our response: Thank you, we have corrected this typo in the revised manuscript.
