# Supplementary material for: Recurrent disease progression networks for modelling risk trajectory of heart failure
Source: PLoS One. 2021 Jan 6;16(1):e0245177. doi: 10.1371/journal.pone.0245177 (PMC7787457; doi:10.1371/journal.pone.0245177)
Supplement: Attachment — Submitted filename: DHTM_PLoS1_Response2.pdf [file pone.0245177.s013.pdf]

Response to Reviewers

Reviewer #1:

The authors' responses and revisions have answered most of my questions. However, there are still some confusions.

1) The authors added a new section, i.e., Section 4.4, to describe the various choices of thresholds, but the notations there are very confusing.

a) The "frequency threshold" was defined as m/(m+k) and was stated in words as the number of positive labels divided by the total number of patients. So, it appears that m is the number of positive labels, i.e., the HF events, and (m+k) is the total number of patients. But these are in contradiction to what were described in the beginning of the section: m is defined as the number of time points for the non-HF events, i.e., the negative labels, and (m+k) is the total number of time points over all the patients (in the training set), not just the total number of patients (in the training set).

Our response: We thank the reviewer for pointing out the typos. We have replaced “number of positive labels divided by the total number of patients” by the correct definition: “number of positive HF-events divided by the total number of **time points over all** patients”. Also, the frequency threshold is defined as **k/(m+k)** because k is the number of positive HF-events and m is the number of non-HF events in the training set. We apologize for the typo.

b) The authors defined p_bar = (p_1, p_2, ... , p_k)/k. Is p_bar is vector or scalar? It looks to me that p_bar, as an average of probabilities, should be defined as (p_1+p_2+...+p_k)/k. The same question exists for n_bar = (n_1, n_2, ... , n_m)/m.

Our response: We thank the reviewer for noticing the typos. We have corrected the definitions of p_bar and n_bar to be respectively $\overline{p} = (p_1 + \cdots + p_k) / k$ and $\overline{n} = (n_1 + \cdots + n_m) / m$

2) For consistency in style, I would suggest rewriting the sentence "Interestingly, the Cox regression achieved the lowest prediction accuracy at 10% AUPRC" (in Section 5.1, 2nd paragraph) as "Interestingly, the Cox regression achieved the lowest AUPRC of 0.1096."

Our response: We thank the reviewer for pointing out this inconsistency. We have modified the manuscript, the updated sentence is now “Interestingly, the Cox regression achieved the lowest AUPRC of 0.1096.”

Reviewer #2:

I thank the authors for their responses. With the new adaptations, I find the article, especially in terms of medical readability, much improved. The clarification of "acute comorbidities" also contributes to this. I only have a few suggestions to improve typo's and a choice of words:

Abstract: space missing between "acute" and "co-morbidities" in the sentence "HF and a set of acuteco-morbidities".

Line 25: space missing between "acute" and "co-morbidities" in the sentence "HF and a set of acuteco-morbidities".

Our response: We thank the reviewer for pointing out this typing mistake and we have corrected it in the updated manuscript.

Line 322: You write "personalized medicine", which is basically a correct term. However, precision medicine is being used more and more often nowadays, because some say that medicine is always "personalized". However, I leave this to the preference of the author.

Our response: We thank the reviewer for the suggestion. We agree completely that precision medicine is the term that is more accurate and used currently. We have replaced mentions of *personalized medicine* with *precision medicine* in the manuscript.
